# Adolescents’ Popularity-Motivated Aggression and Prosocial Behaviors: The Roles of Callous-Unemotional Traits and Social Status Insecurity

**DOI:** 10.3389/fpsyg.2021.606865

**Published:** 2021-01-28

**Authors:** Michelle F. Wright, Sebastian Wachs, Zheng Huang

**Affiliations:** ^1^Department of Psychology, Child Study Center, Pennsylvania State University, University Park, PA, United States; ^2^Faculty of Social Studies, Masaryk University, Brno, Czechia; ^3^Department of Educational Sciences, University of Potsdam, Potsdam, Germany; ^4^National Anti-Bullying Research and Resource Centre, Dublin City University, Dublin, Ireland; ^5^School of Business and Trade, Nanjing Vocational University of Industry Technology, Nanjing, China

**Keywords:** social status insecurity, callousness, unemotional, uncaring, callous-unemotional traits, aggression, cyberaggression, prosocial

## Abstract

As competition over peer status becomes intense during adolescence, some adolescents develop insecure feelings regarding their social standing among their peers (i.e., social status insecurity). These adolescents sometimes use aggression to defend or promote their status. The aim of this study was to examine the relationships among social status insecurity, callous-unemotional (CU) traits, and popularity-motivated aggression and prosocial behaviors among adolescents, while controlling for gender. Another purpose was to examine the potential moderating role of CU traits in these relationships. Participants were 1,047 (49.2% girls; *M*_age_ = 12.44 years; age range from 11 to 14 years) in the 7th or 8th grades from a large Midwestern city. They completed questionnaires on social status insecurity, CU traits, and popularity-motivated relational aggression, physical aggression, cyberaggression, and prosocial behaviors. A structural regression model was conducted, with gender as a covariate. The model had adequate fit. Social status insecurity was associated positively with callousness, unemotional, and popularity-motivated aggression and related negatively to popularity-motivated prosocial behaviors. High social status insecurity was related to greater popularity-motivated aggression when adolescents had high callousness traits. The findings have implications for understanding the individual characteristics associated with social status insecurity.

## Introduction

As competition over peer status becomes intense during adolescence, some adolescents develop insecure feelings regarding their social standing among their peers (i.e., social status insecurity). These adolescents sometimes use relational and physical aggression to defend or promote their status (Li and Wright, 2013) and are not likely to engage in prosocial behaviors (e.g., helping behaviors; Li et al., 2010). Relational aggression is defined as a type of aggression aimed at damaging relationships or social status (Crick and Grotpeter, 1995); physical aggression is defined as threatening physical harm, and includes hitting and kicking (Kaye and Erdley, 2011). With adolescents’ increasing use of information and communication technologies (ICTs), they may also consider these technologies as another way to attain a higher social standing. Supporting this proposal, Wright (2014) found that popularity was positively associated with cyberaggression (i.e., intentionally harassing, embarrassing, outing, excluding, and intimidating others through ICTs).

It is currently unknown whether adolescents with high social status insecurity and callous-unemotional (CU) traits would also use aggressive and prosocial behaviors in the online and offline contexts to gain popularity. In the literature, children with CU traits tend to minimize the extent to which aggression causes the victim suffering and they openly acknowledge caring less about the distress and suffering of others (Pardini and Byrd, 2012). They are less intimidated by the possibility of being punished for aggressive behaviors and often view aggression as an effective means for dominating others. Therefore, children with CU traits may have malicious social schemas and they may use aggressive behaviors to obtain a desired social standing. Thus, the focus of the present research was to examine the relationships among, CU traits, social status insecurity, and popularity-motivated aggression and prosocial behaviors among adolescents, while controlling for gender. Another aim of this research was to examine the potential moderating effect of CU traits in these relationships. Such a proposal is reasonable as CU traits characterize children who are less sensitive to cues of punishment when a reward-oriented response is primed, potentially resulting in them being unconcerned with punishment that may occur after engaging in aggressive behaviors to promote a higher peer status (O’Brien and Frick, 1996). Gaining popularity or a higher social standing among one’s peers using aggressive behaviors in the offline and cyber contexts may present a motivating reward for adolescents with CU traits.

## Callous-Unemotional Traits

According to Frick and colleagues, CU traits are characterized by “a lack of guilt and remorse, a lack of concern for the feelings of others, shallow or superficial expression of emotions, and a lack of concern regarding performance in important activities” (e.g., see review by Frick et al., 2014, p. 533). CU traits are a subtype of antisocial behavior and conduct problems that occur in childhood and endure into adolescence and adulthood (Frick and Dickens, 2006; Frick and White, 2008; Pardini and Frick, 2013). These traits are indicative of the affective features related to psychopathic tendencies and involve the following characteristics: having blunted emotions, problems recognizing distress in others, and having little concern with being punished for misconduct (Hare and Neumann, 2008; Frick et al., 2013). CU traits are associated with unique cognitive and affective characteristics that increase long-term behavioral problems among adolescents (Frick and White, 2008; Salekin and Lynam, 2010). Adolescents with these traits are typically aggressive and have persistent patterns of antisocial behaviors and show reduced responsiveness to teacher discipline and reward strategies (Allen et al., 2016, 2018; Wright et al., 2019).

Researchers conceptualized three dimensions of CU traits, including callousness, uncaring, and unemotional traits (Frick, 2004). Callousness is characterized by a lack of empathy, remorse, and guilt, and having little concern for misconduct. Uncaring is characterized by having little concern for other people and performing tasks (e.g., classwork). Unemotional is characterized by having shallow affect or deficiencies in emotional affect. Many researchers combine these three dimensions into a single dimension of CU traits, and such traits are related to face-to-face bullying perpetration among adolescents (Fanti et al., 2009; Viding et al., 2009). To account for the associations among CU traits, bullying, and aggression, researchers propose that adolescents with these traits lack remorse for their actions and have little empathy for others’ feelings, resulting in less inhibition toward engaging in aggressive acts. Adolescents with CU traits are also less likely to engage in prosocial behaviors (e.g., helping behaviors; Waller and Hyde, 2018).

Other researchers have examined the three dimensions of CU traits separately. Findings from this research indicated that callousness and uncaring traits were correlated positively with face-to-face traditional bullying and cyberbullying perpetration (Essau et al., 2006; Kimonis et al., 2008; Fanti et al., 2009; Roose et al., 2010; Ciucci and Baroncelli, 2014; Kokkinos et al., 2014; Kokkinos and Voulgaridou, 2017). In addition, researchers have also revealed that the uncaring and unemotional dimensions are related negatively to empathy (Kimonis et al., 2008; Roose et al., 2010). The consensus from the literature is usually that the unemotional dimension is unrelated to aggression perpetration (e.g., Kimonis et al., 2008; Roose et al., 2010; Ansel et al., 2015).

## Social Status Insecurity

During adolescence, some adolescents develop concerns or insecure feelings regarding their relative social standing among their peers (LaFontana and Cillessen, 2002). Such concerns may trigger some adolescents to believe that their peer status is not as high as they would like and/or feel threatened by their position in the peer group. Concern and insecurity with their social standing within the peer group is referred to as social status insecurity (Li and Wright, 2013). When concern with peer status increases, some adolescents may defend and/or promote their status by engaging in aggressive behaviors (Sijtsema et al., 2009). Research has revealed positive associations between adolescents’ social status insecurity and their perpetration of physical and relational aggression, as well as negative relationships between social status insecurity and prosocial behaviors (Li et al., 2010; Li and Wright, 2013). Studies on the linkage between social status insecurity and aggression have examined general forms of aggression, unrelated to whether the aggressive behaviors were motivated by the desire to attain a higher peer status, specifically popularity (i.e., Li et al., 2010; Li and Wright, 2013; Wright and Wachs, 2019; Long and Li, 2020). Thus, to better understand whether adolescents with insecure feelings regarding their social standing engage in aggression and use less prosocial behaviors to maintain or attain peer status, follow-up research is needed that focuses on the associations between social status insecurity and popularity-motivated aggressive (i.e., relational, physical, and cyber) and prosocial behaviors. Because social status insecurity involves insecure feelings regarding adolescents’ social standing, understanding their use of popularity-motivated behaviors may address gaps in the literature on the behavioral strategies these adolescents use to improve their peer status in the peer group.

Thus far, no research has focused on characteristics or personality traits that may relate to social status insecurity. Such a focus is important because it can inform literature on understanding which adolescents are most at risk for social status insecurity and aggressive behaviors. Studying CU traits may improve our understanding of how individual differences contribute to the relationships between social status insecurity and aggressive behaviors.

It is currently unknown whether having certain CU traits may increase the positive relationship between social status insecurity and aggression. Given that aggression is often used by adolescents with high social status insecurity, it may be likely that having high levels of callousness and uncaring traits could increase their perpetration of popularity-motivated aggression, especially considering that certain dimensions of CU traits are linked positively to aggression (Essau et al., 2006; Kimonis et al., 2008; Fanti et al., 2009; Roose et al., 2010; Ciucci and Baroncelli, 2014; Kokkinos et al., 2014; Kokkinos and Voulgaridou, 2017). In the literature on this topic, adults with CU traits have a propensity toward seeking social rewards, regardless of whether their actions are perceived as cruel or unkind (Foulkes et al., 2014b). In another study, Foulkes et al., (2014b) found that adult males with psychopathic traits did not endorse affiliative or community relationships and instead valued goals related to their image. Other research has revealed that adolescents with CU traits were more likely to endorse social goals associated with revenge, dominance, and forced respect and less likely to endorse conflict avoidance and friendship building social goals (Pardini, 2011). Thus, adolescents with callousness and uncaring traits may engage in aggressive behaviors to promote their social standing among their peers because they enjoy being cruel to gain social rewards, dominance, and respect, and promote their image (e.g., popularity). The uncaring dimension is not usually related to aggression, but perhaps the effect of this trait on popularity-motivated aggression may be different for adolescents with high social status insecurity, considering their propensity for engaging in aggression. High levels of CU traits are linked to less prosocial behavior, and thus these traits may further diminish the likelihood of adolescents with high social status insecurity engaging in popularity-motivated prosocial behavior.

## The Present Study

The aim of the present study was to examine the associations among social status insecurity, CU traits (i.e., callousness, uncaring, and unemotional), popularity-motivated aggression (i.e., relational, physical, and cyber), and popularity-motivated prosocial behaviors. Another purpose was to investigate the potential moderating effect of CU traits in the relationships between social status insecurity and popularity-motivated aggression and popularity-motivated prosocial behavior. Thus, the study examined the following hypotheses:

*Hypothesis 1*: Social status insecurity is positively related to CU traits.
*Hypothesis 2*: Social status insecurity is positively related to popularity-motivated aggression.
*Hypothesis 3*: Social status insecurity is negatively related to popularity-motivated prosocial behaviors.
*Hypothesis 4*: CU traits are positively related to popularity-motivated aggression.
*Hypothesis 5*: CU traits are negatively related to popularity-motivated prosocial behaviors.
*Hypothesis 6*: CU traits will moderate the relationships among social status insecurity and popularity-motivated aggression.
*Hypothesis 7*: CU traits will not moderate the relationship between social status insecurity and popularity-motivated prosocial behaviors.

## Materials and Methods

### Participants

The participants were 1,047 (49.2% girls; *M*_age_ = 12.44 years; age range from 11 to 14 years) adolescents from seven middle schools. Adolescents were either in the 7th (*n* = 516) or 8th (*n* = 531) grade. The middle schools were located in middle-class suburbs of a large Midwestern city. The majority of adolescents identified as white/Caucasian (70.6%), followed by Latino/a (13.6%), black/African American/Caribbean (6.7%), Asian (2.6%), and Native Hawaiian/Other Pacific Islander (0.3%). Most of the adolescents’ parents/guardians had 2-year or 4-year college degrees (76%), with 21% completing a high school diploma or GED and 3% not completing high school. Approximately 28–46% of adolescents in the middle schools qualified for free or reduced cost lunch.

### Procedures

A list of 160 middle schools surrounding a large Midwestern city was created; of these schools, 10 were randomly selected and recruited for the study. Recruitment letters were emailed to school principals, with three declining to have their schools participate in the study due to other commitments. Meetings were held among the principals, teachers, and research personnel. The purpose of the meeting was to describe the study, explain how adolescents could participate, and describe what adolescents would be expected to do for the study. Next, classroom announcements were made to 7th and 8th grade classrooms. The purpose of the announcement was to describe the study, indicate what they would do, and disseminate a letter and parental permission slip for adolescents to take home to their parents/guardians. A parent questionnaire was sent home as well to adolescents’ parents/guardians. Parents who wanted their child to participate were asked to return the parental permission slip to their child’s school and complete a short demographics questionnaire about their education level and family income. A total of 1,104 letters were sent home, with 1,067 returned. Approximately 1,049 adolescents were given their parents’/guardians’ permission to participate in the study. Questionnaires were administered to adolescents in their school’s computer lab during regular school hours. Prior to completing the questionnaires, adolescents provided their assent. Two adolescents declined to participate, making the final total 1,047. They completed questionnaires on their background information (i.e., age, gender, and ethnicity), social status insecurity, CU traits (callousness, unemotional, and uncaring), and popularity-motivated relational aggression, physical aggression, cyberaggression, and prosocial behaviors. The questionnaires were originally administered to samples of adolescents and demonstrated adequate validity and reliability, except for the CU traits measure. This measure has demonstrated validity and reliability in various samples of adolescents (e.g., Eisenbarth et al., 2016; Truedsson et al., 2019).

### Measures

#### Social Status Insecurity

Adolescents rated their feelings of insecurity concerning their social status and social standing on a scale from 1 (*never*) to 5 (*all the time*; Li et al., 2010; Li and Wright, 2013). There were six items, with examples including: I worry that my peers do not like me, I feel that my status among my peers is not high, and I worry about my popularity among my peers. Cronbach’s alpha was 0.84.

#### Callous-Unemotional Traits

Adolescents completed the 24-item Inventory of Callous-Unemotional Traits (Frick, 2004). Items were rated on a scale from 0 (*not at all true*) to 3 (*definitely true*). The questionnaire includes three subscales, including callousness (11 items; e.g., The feelings of others are unimportant to me), unemotional (eight items; e.g., I hide my feelings from others), and uncaring (five items; e.g., I try not to hurt others’ feelings – reversed scored). Items from each subscale were averaged separately to form scores on callousness, unemotional, and uncaring traits. Cronbach’s alphas were 0.83 for callousness, 0.81 for unemotional, and 0.86 for uncaring.

#### Popularity-Motivated Aggression and Prosocial Behavior

Adolescents answered 17-items regarding their popularity-motivated relational aggression, physical aggression, cyberaggression, and prosocial behaviors. This questionnaire was adapted from the peer-nominations for aggressive and prosocial behaviors questionnaire (Crick and Grotpeter, 1995). The directions were revised to ask adolescents to rate the behaviors according to how often they have engaged in a particular behavior to increase their popularity among their peers within the current school year (Wright, 2020). There were five items used for popularity-motivated relational aggression (e.g., How often do you keep a peer out of a group of other peers because you are mad at him/her?), three items for physical aggression (e.g., How often do you hit other peers?), five items for cyberaggression (e.g., How often do you call other peers mean and hurtful names online or through text messages), and four items for prosocial behaviors (e.g., How often do you help your peers?). Items were rated on a scale from 1 (*never*) to 5 (*all of the time*). Cronbach’s alphas were 0.88 for relational aggression, 0.83 for physical aggression, 0.85 for cyberaggression, and 0.83 for prosocial behaviors.

### Analytic Plan

To examine the measurement model, a confirmatory factor analysis was conducted using *Mplus 7.11*. The fit of the measurement model was adequate, *χ*^2^ = 366.67, *df* = 382, *p* < n.s., CFI = 0.99, TLI = 0.98, RMSEA = 0.04, SRMR = 0.05. All standard factor loadings were significant (*ps* < 0.001), ranging from 0.69 to 0.89. The items served as indicators for the latent variables for the structural regression model, which was used to test the study’s hypotheses. For the structural regression model, paths were specified from social status insecurity to callousness, uncaring, and unemotional traits, from social status insecurity to popularity-motivated relational aggression, physical aggression, cyberaggression, and prosocial behaviors. Paths were also added from social status insecurity to popularity-motivated relational aggression, physical aggression, cyberaggression, and prosocial behaviors. Gender was controlled by allowing it to predict social status insecurity, CU traits, and popularity-motivated aggressive and prosocial behaviors. Two-way interactions were included between social status insecurity and callousness, social status insecurity and uncaring, social status insecurity and unemotional. Significant interactions were examined using the interaction program, which provides visual representation of the unstandardized sample regression slopes and the simple slopes at +1 SD, the mean, and −1 SD.

## Results

Prior to examining the study’s hypotheses, descriptive statistics and correlations were conducted for all variables (see Table 1). The correlations indicated that social status insecurity was related positively to callousness, unemotional, and popularity-motivated relational aggression, physical aggression, and cyberaggression. Social status insecurity was related negatively to popularity-motivated prosocial behaviors and unrelated to uncaring traits. Callousness was associated with unemotional, and popularity-motivated relational aggression, physical aggression, and cyberaggression, as well as related negatively to popularity-motivated prosocial behaviors and unrelated to uncaring. Uncaring was unrelated to popularity motivated relational aggression, physical aggression, cyberaggression, and prosocial behaviors. Popularity-motivated relational aggression was related positively to popularity-motivated physical aggression and prosocial behaviors. Popularity-motivated physical aggression was associated positively with popularity-motivated cyberaggression and prosocial behaviors. Popularity-motivated cyberaggression was related positively to popularity-motivated prosocial behaviors.

**Table 1:** Correlations among variables.

S. No.		1	2	3	4	5	6	7	8
1.	SSI	---							
2.	Callousness	0.27^**^	---						
3.	Unemotional	0.22^*^	0.26^**^	---					
4.	Uncaring	0.10	0.18^*^	0.16^*^	---				
5.	PM RA	0.33^***^	0.30^***^	0.22^*^	0.10	---			
6.	PM PA	0.30^***^	0.27^**^	0.20^*^	0.09	0.29^***^	---		
7.	PM CA	0.28^***^	0.24^**^	0.24^**^	0.11	0.31^***^	0.31^***^	---	
8.	PM PB	−0.24^**^	−0.22^*^	−0.20^*^	−0.11	−0.26^**^	−0.25^**^	−0.21^*^	---

SSI, social status insecurity; PM, popularity-motivated; RA, relational aggression; PA, physical aggression; CA, cyberaggression; PB, prosocial behaviors.

* *p* < 0.05;

** *p* < 0.01;

*** *p* < 0.001.

The results suggested that the structural model fit the data adequately, *χ*^2^ = 508.35, *df* = 400, *p* < 0.001, CFI = 0.98, TFL = 0.98, RMSEA = 0.05, SRMR = 0.04 (see Table 2). Social status insecurity was related positively to callousness (*β* = 0.16, *p* < 0.05) and unemotional (*β* = 0.15, *p* < 0.05) but was unrelated to uncaring (*β* = 0.03, *p* = n.s.). Callousness and unemotional were related positively to popularity-motivated relational aggression (callousness: *β* = 0.27, *p* < 0.001; unemotional: *β* = 0.16, *p* < 0.05), physical aggression (callousness: *β* = 0.23, *p* < 0.05; unemotional: *β* = 0.16, *p* < 0.05), and cyberaggression (callousness: *β* = 0.27, *p* < 0.001; unemotional: *β* = 0.20, *p* < 0.05), but negatively associated with popularity-motivated prosocial behaviors (callousness: *β* = −0.24, *p* < 0.05; unemotional: *β* = −0.18, *p* < 0.05). Uncaring was unrelated to popularity-motivated relational aggression (*β* = 0.03, *p* = n.s.), physical aggression (*β* = 0.02, *p* = n.s.), cyberaggression (*β* = 0.04, *p* = n.s.), and prosocial behaviors (*β* = −0.10, *p* = n.s.). Social status insecurity was associated positively with popularity-motivated relational aggression (*β* = 0.28, *p* < 0.001), physical aggression (*β* = 0.13, *p* < 0.05), and cyberaggression (*β* = 0.26, *p* < 0.01), but negatively related to popularity-motivated prosocial behaviors (*β* = −0.20, *p* < 0.05). Gender was unrelated to all variables examined in this study.

**Table 2:** Results from the structural regression model.

	Callousness				Unemotional				Uncaring							
Predictors	*b*	*β*	*SE*	*p*	*b*	*β*	*SE*	*p*	*b*	*β*	*SE*	*p*				
SSI	**0.09**	**0.16**	**0.03**	**0.031**	**0.10**	**0.15**	**0.04**	**0.030**	0.01	0.03	0.01	0.767				
	Popularity-motivated relational aggression				Popularity-motivated physical aggression				Popularity-motivated cyber aggression				Popularity-motivated prosocial behaviors			
Predictors	*b*	*β*	*SE*	*p*	*b*	*β*	*SE*	*p*	*b*	*β*	*SE*	*p*	*b*	*β*	*SE*	*p*
SSI	**0.15**	**0.32**	**0.08**	**0.001**	**0.11**	**0.27**	**0.06**	**0.009**	**0.11**	**0.27**	**0.07**	**0.008**	**−0.10**	**−0.20**	**0.04**	**0.026**
Callousness	**0.12**	**0.27**	**0.07**	**0.001**	**0.10**	**0.23**	**0.06**	**0.029**	**0.10**	**0.27**	**0.06**	**0.001**	**−0.13**	**−0.24**	**0.05**	**0.019**
Unemotional	**0.04**	**0.16**	**0.04**	**0.021**	**0.03**	**0.16**	**0.04**	**0.041**	**0.05**	**0.20**	**0.04**	**0.031**	**−0.09**	**−0.18**	**0.04**	**0.030**
Uncaring	0.01	0.03	0.01	0.871	0.01	0.01	0.01	0.919	0.01	0.04	0.01	0.841	−0.01	−0.10	0.02	0.661
Interactions	*b*	*β*	*SE*	*p*	*b*	*β*	*SE*	*p*	*b*	*β*	*SE*	*p*	*b*	*β*	*SE*	*p*
SSI × callousness	**0.01**	**0.10**	**0.03**	**0.033**	**0.04**	**0.09**	**0.03**	**0.037**	**0.05**	**0.12**	**0.04**	**0.023**	−0.01	−0.01	0.01	0.921
SSI × unemotional	0.01	0.01	0.01	0.901	0.01	0.02	0.02	0.891	0.01	0.01	0.01	0.916	−0.01	−0.03	0.01	0.864
SSI × uncaring	−0.01	−0.01	0.01	0.924	0.01	0.01	0.01	0.931	0.01	0.02	0.01	0.888	−0.02	−0.02	0.01	0.896

Bolded numbers indicate significant findings. SSI, social status insecurity.

Significant interactions were found between social status insecurity and callousness. Probing the interaction further revealed that adolescents with higher social status insecurity reported higher popularity-motivated relational aggression (*B* = 0.16, *SE* = 0.08, *p* < 0.01 at +1 *SD*), physical aggression (*B* = 0.12, *SE* = 0.05, *p* < 0.05 at +1 *SD*), and cyber aggression (*B* = 0.14, *SE* = 0.06, *p* < 0.01 at +1 *SD*) when they had higher levels of callousness. The simple slopes were not significant for mean or lower levels of callousness. The other interactions for unemotional and uncaring were not significant.

## Discussion

The purpose of this research was to examine the moderating effect of CU traits on the associations between social status insecurity and popularity-motivated aggression and prosocial behaviors. This study addressed an important gap in the literature regarding the influence of CU traits on popularity-motivated aggressive and prosocial behaviors among adolescents with high social status insecurity. The findings revealed that social status insecurity was positively related to callousness and unemotional traits, popularity-motivated relational aggression, physical aggression, and cyberaggression, but negatively associated with prosocial behaviors. Furthermore, high levels of callousness strengthened the positive relationship between social status insecurity and popularity-motivated aggression.

To our knowledge, no studies have examined the characteristics associated with social status insecurity. To this end, we examined the correlation between social status insecurity and the three dimensions of CU traits, including callousness, unemotional, and uncaring traits. Although research on the characteristics or personality traits associated with social status insecurity is in its infancy, feeling that one’s social standing is threatened in the peer group may be a symptom of the negative characteristics associated with CU traits. In addition, it may also be likely that adolescents with CU traits have a greater desire to improve their social standing as a means of gaining control and increase opportunity for manipulating their peers (Pardini, 2011). We found that social status insecurity was associated positively with callousness and unemotional, but not uncaring traits, partially supporting Hypothesis 1. The characteristics of callousness (e.g., lacking empathy, remorse, and guilt) and unemotional (e.g., shallow or deficient emotional affect) may increase the propensity toward acting negatively toward one’s peers, which could diminish their social standing and increase their insecurity with their status (Hare and Neumann, 2008; Salekin and Lynam, 2010; Frick et al., 2013). Thus, adolescents with callousness and unemotional traits may take action to improve their status, such as engaging in popularity-motivated aggression. Our results provide support for this proposal by finding positive associations between CU traits, specifically, callousness and unemotional, and popularity-motivated aggression (Hypothesis 4; i.e., relational, physical, and cyber) and negative associations between CU (i.e., callousness and unemotional) and popularity-motivated prosocial behaviors (Hypothesis 5). Uncaring traits involve little concern for other people, and this is not characteristics of adolescents with high social status insecurity, who are concerned with their social standing which involves their status in relation to others and the perceptions others have regarding these adolescents’ peer status (Frick, 2004).

Understanding social status insecurity is important because some adolescents develop insecure and concerned feelings regarding their current social standing (Li and Wright, 2013). They may believe that their social standing is either threatened or is not as high as they like, especially when competition in the peer group increases. Consistent with the literature, social status insecurity in the present study was related positively to popularity-motivated relational aggression, physical aggression, and cyberaggression (Hypothesis 2; Li et al., 2010; Li and Wright, 2013). Furthermore, social status insecurity was related negatively to popularity-motivated prosocial behavior (Hypothesis 3), which is consistent with the literature revealing a negative relationship between social status insecurity and peer-nominated prosocial behaviors (Li et al., 2010; Li and Wright, 2013). Our findings further extend the literature to include not only general forms of aggressive and prosocial behaviors but also behaviors carried out specifically to gain a higher social standing in the peer group, specifically greater popularity.

We found two-way interactions between social status insecurity and callousness for popularity-motivated relational aggression, physical aggression, and cyberaggression, providing some support for Hypothesis 6. More specifically, high levels of callousness increased the positive relationship between social status insecurity and popularity-motivated aggression. None of the other traits moderated this relationship. Furthermore, moderation effects were not found for popularity-motivated prosocial behaviors (Hypothesis 7). It is difficult to determine if this finding is aligned with the literature because of the lack of research. CU traits are linked to social rewards, regardless of whether one’s actions are perceived as cruel or unknown, less affiliative or community relationship goals, image-based values, and social goals associated with revenge, dominance, and forced respect (Pardini, 2011; Foulkes et al., 2014a,b). Because adolescents with high social status insecurity engage in popularity-motivated aggression and can also have callousness traits, they may have a greater propensity toward aggressive behaviors to gain status because of their desire for social rewards related to social status or dominance and maintaining image-based values, their insecure feelings regarding their social standing, and their lack of empathy, guilt, and remorse (Hare and Neumann, 2008; Salekin and Lynam, 2010; Frick et al., 2013).

### Limitations and Future Directions

Our findings providing valuable insight into adolescents’ insecurity with their social standing in the peer group, the characteristics associated with this insecurity, and their utilization of aggression to improve their status. However, we want to acknowledge some limitations of the present research to further the development of research on social status insecurity. First, the directions of the associations between all variables is unknown, given the cross-sectional nature of the study. Follow-up research should be conducted with longitudinal designs to better understand the temporal ordering of the associations between social status insecurity, CU traits, and popularity-motivated aggression. Second, we considered social status insecurity regarding adolescents’ current social standing, but we did not consider the likelihood that adolescents may be concerned with different types of popularity. Future research may consider specific types of social status insecurity related to popularity (often linked to aggressive behaviors) and social preference (often linked to prosocial behaviors). Third, we relied on a single informant for the variables in this study which could have led to issues with shared method variance. Follow-up research should incorporate multiple informants, such as teachers, parents, and peers, to assess the variables included in this study. Furthermore, future research could also incorporate alternative methods for assessing the variables in this study, including experimental designs and peer observation.

### Conclusion

The purpose of this study was to examine the relationships between social status insecurity, CU traits, and popularity-motivated aggression and prosocial behaviors, as well as the moderation of CU traits in the relationships between social status insecurity and popularity-motivated aggression and prosocial behaviors. This study was one of the first to examine CU traits as personality correlates of social status insecurity. It was only one of the first studies to delineate aggression and prosocial behaviors as being carried out to gain increased popularity in the peer group (Wright, 2020). Findings of this study contribute to our understanding of the characteristics associated with social status insecurity and their perpetration of aggressive behaviors. These findings potentially contribute to the development of intervention programs designed to reduce adolescents’ popularity-motivated aggression. It may also be possible to identify adolescents at risk for engaging in popularity-motivated aggression based on their levels of social status insecurity and CU traits.

## Data Availability Statement

The datasets presented in this article are not readily available because of IRB restrictions. Requests to access the datasets should be directed to MW, mfw5215@psu.edu.

## Ethics Statement

The studies involving human participants were reviewed and approved by DePaul University, #MW08021 OPSY. Written informed consent to participate in this study was provided by the participants’ legal guardian/next of kin.

## Author Contributions

MW designed this study, drafted the manuscript, and performed the statistical analyses. SW and ZH provided constructive and editorial feedback on drafts of the manuscript. All authors read and approved the manuscript.

### Conflict of Interest

The authors declare that the research was conducted in the absence of any commercial or financial relationships that could be construed as a potential conflict of interest.
